# In Vivo Simulation of Magnesium Degradability Using a New Fluid Dynamic Bench Testing Approach

**DOI:** 10.3390/ijms20194859

**Published:** 2019-09-30

**Authors:** Ole Jung, Dario Porchetta, Marie-Luise Schroeder, Martin Klein, Nils Wegner, Frank Walther, Frank Feyerabend, Mike Barbeck, Alexander Kopp

**Affiliations:** 1 Department of Oral Maxillofacial Surgery, University Medical Center, 20246 Hamburg-Eppendorf, Germany; mari.schroeder@uke.de (M.-L.S.); mike.barbeck@icloud.com (M.B.); 2 Department of Materials Test Engineering (WPT), TU Dortmund University, 44227 Dortmund, Germany; dario.porchetta@meotec.eu (D.P.); martin.klein@tu-dortmund.de (M.K.); nils.wegner@tu-dortmund.de (N.W.); frank.walther@tu-dortmund.de (F.W.); 3 Meotec GmbH, 52068 Aachen, Germany; alexander.kopp@meotec.eu; 4 Institute of Materials Research, Division Metallic Biomaterials, Helmholtz-Zentrum Geesthacht, 21502 Geesthacht, Germany; frank.feyerabend@hzg.de; 5 BerlinAnalytix GmbH, 12109 Berlin, Germany

**Keywords:** bioreactor, magnesium, degradation, plasma electrolytic oxidation (PEO)

## Abstract

The degradation rate of magnesium (Mg) alloys is a key parameter to develop Mg-based biomaterials and ensure in vivo-mechanical stability as well as to minimize hydrogen gas production, which otherwise can lead to adverse effects in clinical applications. However, in vitro and in vivo results of the same material often differ largely. In the present study, a dynamic test bench with several single bioreactor cells was constructed to measure the volume of hydrogen gas which evolves during magnesium degradation to indicate the degradation rate in vivo. Degradation medium comparable with human blood plasma was used to simulate body fluids. The media was pumped through the different bioreactor cells under a constant flow rate and 37 °C to simulate physiological conditions. A total of three different Mg groups were successively tested: Mg WE43, and two different WE43 plasma electrolytically oxidized (PEO) variants. The results were compared with other methods to detect magnesium degradation (pH, potentiodynamic polarization (PDP), cytocompatibility, SEM (scanning electron microscopy)). The non-ceramized specimens showed the highest degradation rates and vast standard deviations. In contrast, the two PEO samples demonstrated reduced degradation rates with diminished standard deviation. The pH values showed above-average constant levels between 7.4–7.7, likely due to the constant exchange of the fluids. SEM revealed severe cracks on the surface of WE43 after degradation, whereas the ceramized surfaces showed significantly decreased signs of corrosion. PDP results confirmed the improved corrosion resistance of both PEO samples. While WE43 showed slight toxicity in vitro, satisfactory cytocompatibility was achieved for the PEO test samples. In summary, the dynamic test bench constructed in this study enables reliable and simple measurement of Mg degradation to simulate the in vivo environment. Furthermore, PEO treatment of magnesium is a promising method to adjust magnesium degradation.

## 1. Introduction

In medicine, the right choice of biomaterial in an important aspect for the overall success of a surgical therapy. Throughout the years, biomaterials have been used in the field of medicine to enhance the outcome and quality of a procedure. For instance, bony defects require a biomaterial that mimics the characteristics of bone, such as magnesium, whereas the regeneration of soft tissue demands fewer rigid biomaterials, such as polymers and their derivatives [1,2,3,4].

Of the various fields of medicine demanding reliable biomaterials, the fields of orthopedic and maxillofacial surgery, in particular, have been experimenting with different biomaterials to reach an optimal outcome [5,6,7]. In addition to the very commonly used metals, stainless steel and titanium, the degradable metal Mg gets more and more attention [1,8,9,10]. Magnesium is an important physiological element in the human body and can be found in skeletal bones, muscles, soft tissues and in the extracellular compartments as an important regulator of physiological processes [11,12,13]. It is further similar to natural bone in terms of not only inherent mechanical and structural properties, but also biological response, such as osteoconduction or osteopromotion [9]. Since Mg is biodegradable and soluble in physicochemical fluids, the application of Mg-based materials for different surgical procedures (e.g., osteosynthesis, screws for surgical applications) is highly desirable and can avoid secondary implant removal [2,9]. However, the low corrosion resistance of Mg in aqueous solutions can lead to the evolution of hydrogen gas and a pH-increase, which can counteract proper healing of hard and soft tissues [14,15]. In aqueous solution, magnesium reacts according to the following equation [16]:

Mg + 2H_2_O → Mg(OH)_2_ + H_2_ (1)

Thereby, the choice of a suitable magnesium alloy plays an important role. Various alloying elements make it possible to increase the corrosion resistance of magnesium in aqueous solutions [1,17,18,19]. Further improvements are possible using different coatings or geometrical modifications (e.g., plasma electrolytic oxidation (PEO), scaffold structures) [20,21,22,23].

Traditionally, magnesium degradation is measured by physicochemical methods, such as potentiodynamic polarization, electrochemical impedance spectroscopy or scanning electron microscopy (SEM) [24,25,26,27,28,29,30,31,32,33,34,35,36]. More recently, biological methods have been introduced, such as static immersion tests with H_2_ evolution measurement, weight loss measurement of specimens or the microscopic comparison of magnesium degradation in vitro-in vivo under cyclic stress [30,37,38,39,40,41,42,43,44,45]. Thereby, the choice of the corrosive medium has a major influence on the overall corrosion behavior [26,27,42,43,46,47,48,49,50]. However, the measurement of the H_2_ release represents a major role for tissue regeneration, since hydrogen gas generated from Mg degradation can lead to swelling, formation of blisters, organ compression and high levels pH [14,15].

In this study, we developed a simple, yet effective dynamic test bench with several attached bioreactor cells that captures and measures the evolution of H_2_ gas during Mg degradation in a simulated physiological environment under constant flow rate and medium exchange. The Mg alloy WE43 and two PEO variants of WE43 were included as test samples. For comparison, Mg degradation was also monitored using Potentiodynamic polarization (PDP), scanning electron microscopy (SEM) microscopy, cytocompatibility and pH measurement.

## 2. Results

### 2.1. Test Bench and Bioreactor Construction

**Figure 1:**
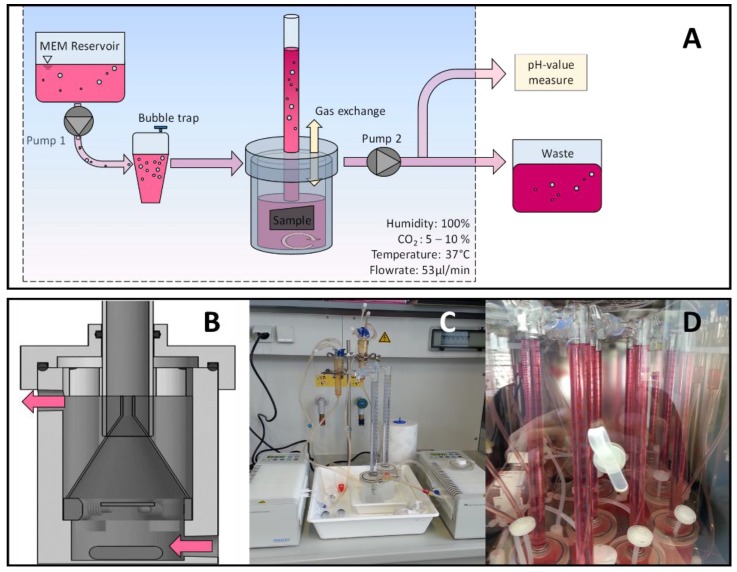
Bioreactor design. (**A**) Each bioreactor cell was independently connected through Versilic silicon tubes to tworeservoirs filled with minimum essential medium (MEM) (1). The medium was pumped by a multichannel roller pump (2) to each bioreactor (3). To avoid the falsification of measurents by foreign gas bubbles in the system, a bubble trap was installed between the first pump and the bioreactor itself (4). A second pump (5), in sync with the first one, allows for the removal of MEM from the degradation chamber towards a waste reservoir (6). Before the second pump, a split line allows for the aliquotation of small volumes of MEM for pH measurements (7). In order to reduce the risk of medium contamination, a PTFE filter acts as a particle barrier between the two compartments (8). (**B**) Schematic illustration of a bioreactor. The flow of the medium is shown by the direction of the arrows and the holder for the specimens is encircled. (**C, D**) Illustrative photographs of the bioreactor cells before (**C**), and throughout the experiment (**D**).

The construction of the test bench with connected bioreactor cells was successfully conducted and according specimens were realized. In total, ten bioreactor cells were operated separately and combined to form a fluid-dynamic testing bioreactor system (Figure 1A–D). The dynamic flow conditions were chosen according physiological model and resulted in a 47% daily exchange of total fluid, which was collected separately and aliquotated for pH measurement. The evolved hydrogen gas from Mg degradation was captured by an upside-down funnel and measured in a scaled cylinder placed over the funnel. Different specimens of the three variants were tested and examined in the bioreactor system, showing a pronounced difference in degradation behavior.

### 2.2. H_2_- and pH-determination

**Figure 2:**
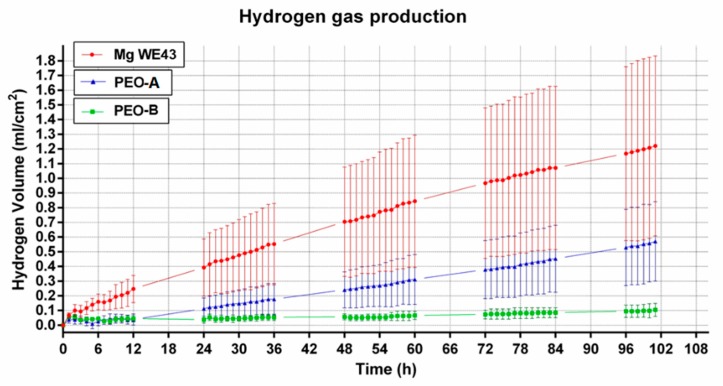
Comparison of the H_2_ gas evolution of PEO-modified and unmodified test samples. The evolution of hydrogen gas varied less for the two PEO ceramiced samples compared to the untreated samples. PEO-B showed an almost linear curve shape and performed better in terms of degradation resistance than PEO-A.

**Figure 3:**
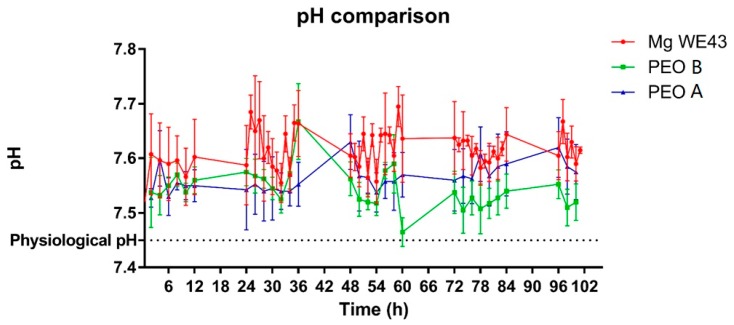
Measurement of the pH within the different cells linked to the degradation rate; time-dependent pH values for Mg WE43, PEO-B and PEO-A samples are shown in the graph. Values are displayed as means ± SD.

The development of hydrogen gas volume of the alloy WE43 increased linearly over time (Figure 2). The rate of the increase was nearly constant over time. H_2_-release rates differed largely among different reactors, which resulted in high standard deviations. The ceramized test samples PEO-A and PEO-B showed a similar and linear increase in hydrogen gas development over time. The mean curves of both PEO samples showed a lower H_2_-release compared to the non-ceramized test samples, which was significantly lower for both ceramized test samples over the first day (*p* < 0.05), before turning insignificant for PEO-A. In comparison, PEO-B showed a rather stable and reduced level of hydrogen gas evolution throughout the 100 h duration of the experiment. After 24 h, mean gas evolution values from PEO-B were significantly different compared to PEO-A. (*p* < 0.05 or less). Overall, the PEO specimens demonstrated low to no deviation in terms of the measured H_2_-values.. The pH values for all samples ranged from 7.4–7.7, most likely due to the continuous medium exchange. However, the untreated alloy showed insignificant higher values over time, which might be related to the increased degradation rate of the unprotected, yet reactive metal (Figure 3).

### 2.3. Surface Topography of the Mg Specimens

**Figure 4:**
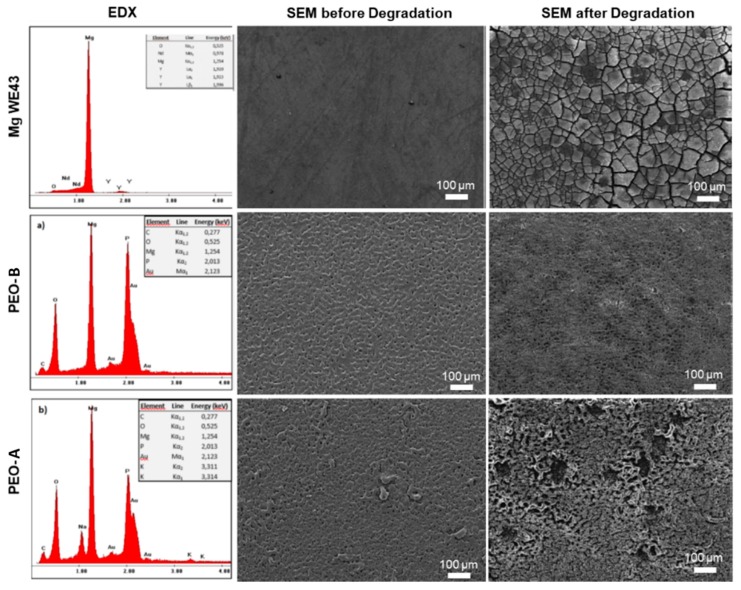
Scanning electron microscopy (SEM) of the surfaces of the Mg specimens (WE43, PEO-B and PEO PEO-A) before and after degradation, revealing a accelerated corrosion attack of the Mg WE43, followed by PEO-A and PEO-B showing almost no change in surface morphology. For the surface of the unprotected Mg WE 43 specimens a smooth surface can be observed, whereas the degradation resulted in a corrosion layer comprising cracks and fissures. In contrast, the PEO treated samples show a micro-structured surface with a microporosity. After degradation, the cavities of the PEO microstructure became more pronounced, while fissures appeared and the surface of the PEO modification turn into a comb-like surface structure with single pittings. These features were significantly more apparent on the PEO PEO-A samples.

Figure 4 shows the specimen surfaces before and after the degradation experiments. Before testing, Mg WE43 test samples revealed a smooth surface, showing no topographical structure except single grooves from the mechanical preparation procedure. In contrast, the PEO treatment introduced microscopically visible patterned structures onto the surfaces of both PEO-B and PEO-A, while PEO-A showed irregularities in morphology depicted by small protrusions of the ceramic filiforms. After degradation, the Mg WE43 specimens exhibited a pronounced uniform layer, showing severe cracks and fissures. While after degradation both PEO variants showed fewer signs of corrosion than the unprotected WE43, PEO A showed both, a visible uniform attack on the microstructure and distinct pittings protruding the PEO surface layere. In contrast, PEO-B almost retained its initial topography with only an uniform and reduced deepening of the microporosity.

### 2.4. PDP Measurements

Table 1 displays the degradation rates in µm/year, which were calculated using the equation by Stern and Geary [50,51]. Thereby, the untreated magnesium test samples showed a manifold higher degradation rate compared to the PEO-treated test samples.

### 2.5. Cytocompatibility and LIVE/DEAD Staining

**Figure 5:**
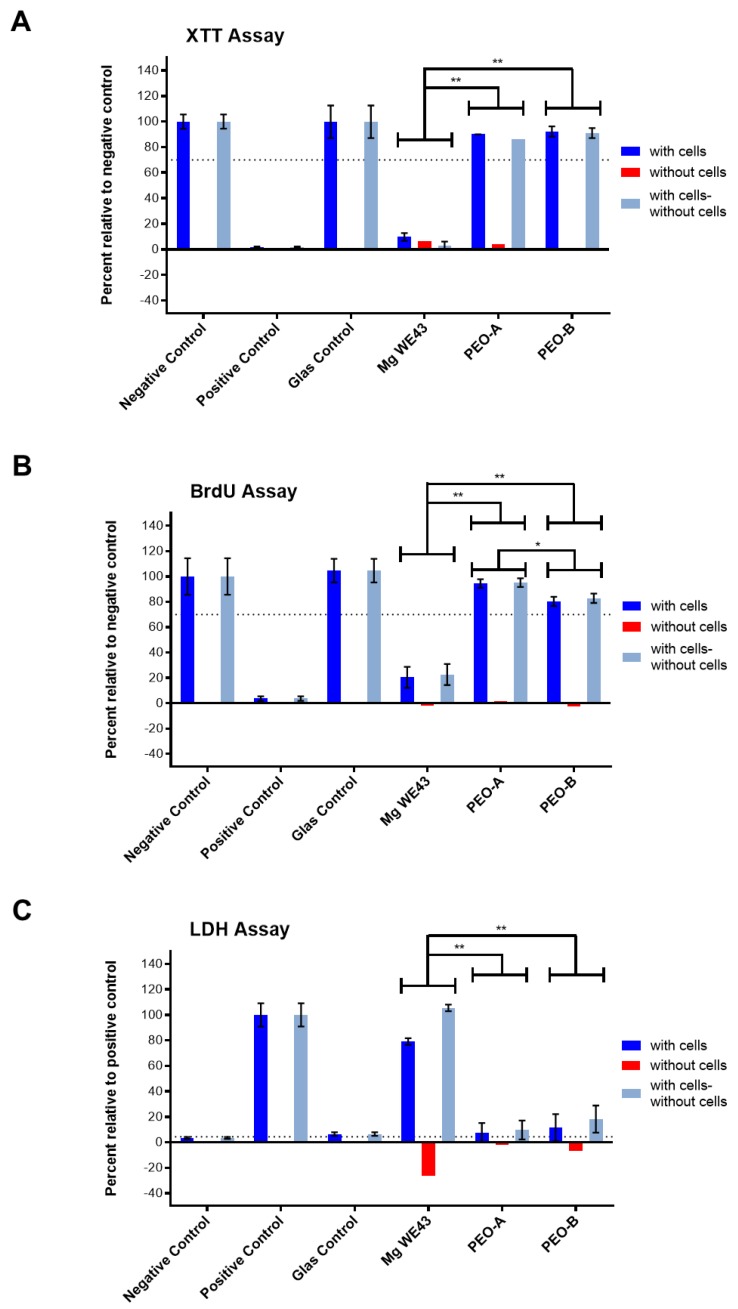
Cytocompatibility assessment of the Mg specimens WE43, PEO-B and PEO-A. (**A**) XTT assay indicating cell viability; (**B**) BrdU assay indicating cell proliferation; (**C**) LDH assay indicating the cytotoxicity of the material. Mg WE43 shows the greatest release of cytotoxic agents; accordingly, low cellular viability and proliferation can be observed. Both, PEO-B and PEO PEO-A demonstrate givenoverall cytocompatibility as depicted by the significant elevation of viability and proliferation values, complemented by a minor release of cytotoxic substances during degradation. Asterixis mark significant differences (* *p* ≤ 0.05, ** *p* ≤ 0.01).

**Figure 6:**
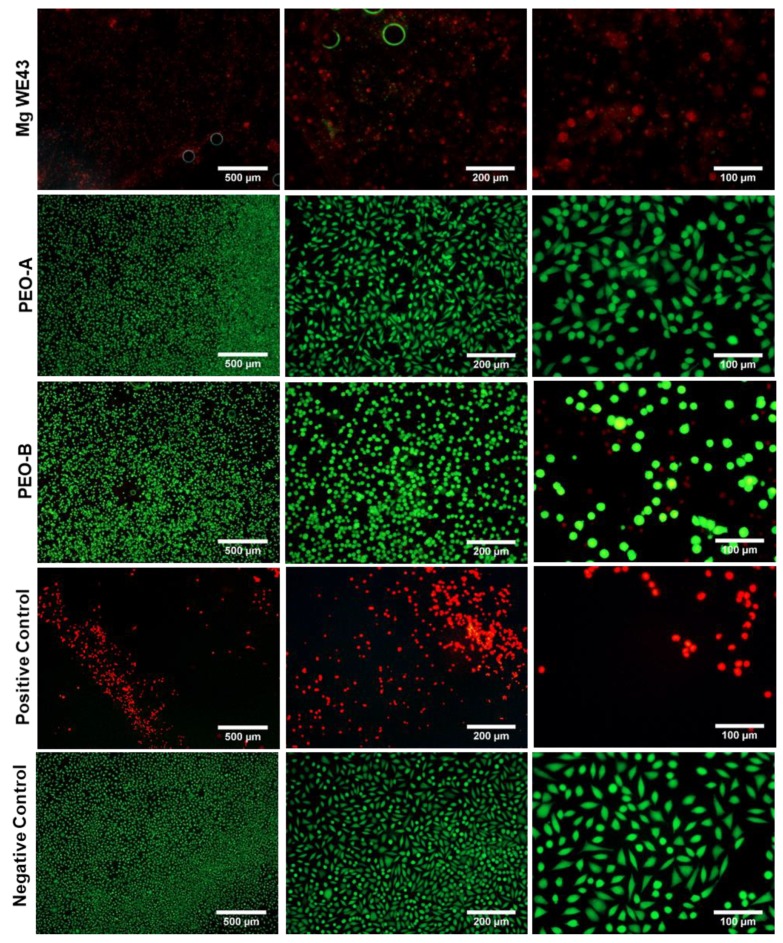
Live/dead staining of L-929-fibroblasts on the surfaces of Mg WE43, PEO-B and PEO-A. Green- and red-stained cells denote viable and non-viable cells, respectively. Cells populating PEO-B specimens showed the highest percentage of viable (green) cells, while those cultured on the Mg WE43 specimens exhibited the lowest viability.

In the XTT, BrdU and LDH-assays, the untreated WE43 alloy showed a cytotoxic effect, comparable to the positive control (Figure 5). In concordance, gas bubbles and nearly no cellular viability or cell proliferation could be detected (Figure 6). The two ceramized surfaces PEO-B and PEO-A exhibited no cytocompatibility indicated by enhanced viability, proliferation and metabolic activity of the tested cell culture, being comparable to the negative control (Figure 5). Thereby, values for PEO-A and PEO-B were highly significantly different from Mg WE43 in all assays (*p* < 0.01). In the BrdU assay, PEO-A showed higher values compared to PEO-B (*p* < 0.05). Live/Dead staining revealed a large number of viable green cells were visible on both ceramized specimens (Figure 6). The cells on PEO PEO-B and the negative control showed a spindle-shaped morphology as a sign of cellular attachment, whereas cells on PEO-A showed rounded, not adherent, morphology.

## 3. Materials and Methods

### 3.1. Mg Variants

A Mg-Y-RE-Zr based magnesium alloy (WE43MEO, Meotec GmbH, Aachen, Germany) nominally containing app. 4% yttrium, 3% Rare Earths and less than 0.6% zirconium with magnesium being the remainer was included in this study. Samples were cut-grinded from a rod (Secotom, Struers GmbH, Willich, Germany), followed by chemical polishing in nitric acid (HNO_3_) (65% in volume). The samples with a final diameter of 18.8 mm and 0.2 mm height were washed in water and ethanol and preserved in a dry-air closet to prevent further oxidation. Prior to the degradation experiments, test samples were degreased in an ultrasonic bath of ethanol for 10 min. Mg WE43 samples further underwent PEO treatment in a stainless-steel container forming the counter-electrode. A layer thickness of 9 +/− 6 µm was targeted for both PEO variants by adjusting current density between 1.35–3.5 A/dm^2^ and the corresponding treatment time between 8–25 min. As electrolytes, two phosphate-rich electrolytes supplemented with different conductivity agents were used (Table 2). For each experiment, *n* = 11 test samples of Mg WE43 and the two types of PEO treatments (PEO-A and PEO-B) were used each.

### 3.2. Test Bench and Bioreactor Design

A test bench allowing for simulated body fluids (medium) to embrace and stream over the different Mg test specimens was designed and realized by conventional manufacturing methods (Figure 1).

Due to its similar chemical composition and ability to simulate human blood plasma, MEM (minimum essential medium) supplemented with 10% fetal bovine serum, penicillin/streptomycin (100 U/mL each) (all from Life Technologies, Carlsbad, CA, USA) with 4 mM *L*-glutamine (Sigma-Aldrich, St. Louis, MO, USA) was chosen as both, degradation and cell culture medium. Comparisons of different culture mediums to simulate in vivo conditions, hence suitable for degradation experiments on magnesium, were published by the authors in advance [50,52]. The whole set of the dynamic testing cells was placed in an incubator at a constant temperature of 37 °C, 6% CO_2_ and an 85%–95% humidity (further referred as to as physiological conditions).

The different cells of the bioreactor comprised a corrosion chamber, in which the specimens were fixed to a holder in order to expose both sides of the discs to the medium stream. Two synchronized multichannel roller pumps (Ismatec^®^ IPC, Wertheim, Germany) were used before and after the corrosion chamber to enable the flow. Hence, each bioreactor was constantly and independently feed with fresh medium through versilic silicon tubing (Biologic inert USP XXIII Class 6) having a flow rate of 53 μL/min, which equals physiological interstitial flow. A bubble trap was used immediately in advance to the entrance of the individual corrosion cells, thereby eliminating foreign bubbles gassing off peripheral components and potentially falsifying the volumetric measurements.

A bifurcation in the tubing leading away from the corrosion cells allowed for aliquotation of small volumes of the used MEMto measure the pH before discarding the medium to the waste reservoir.

Within the corrosion cells, an upside-down funnel was placed in immediate adjacency to the sample in order to capture the evolving hydrogen gas during specimen degradation. The hydrogen gas was further guided into a graduated burette to determine the exact amount of accumulated gas measurement. pH buffering was guaranteed by a open gas exchange between the individual corrosion cells and the incubator’s CO_2_ atmosphere. In order to reduce the risk of medium contamination, a sterile PTFE filter acted as a particle barrier between the different compartments.

All corrosion cells were initially calibrated by injecting 10 mL of O_2_ to the bottom of a single cell followed by measuring the released gas volume in the burette. The degradation experiment was limited to a duration of 100 h total. A total number of 10 bioreactor cells were manufactured and operated simultaneously. Every experiment tested two single variants, each in 4 bioreactor cells. The remaining two corrosion cells served as additional controls to measure false gas evolution from the plain medium within the cells and was used to adjust the measurement results.

After the degradation experiment, specimens were washed in water and ethanol, followed by preservation in a dry cabinet to avoid further degradation until further characterization (PDP, SEM) at a later time point.

### 3.3. Surface Characterization of Mg Specimens

Before and after degradation experiments, surfaces of all utilized Mg specimens were facilitating a scanning electron microscope (SEM/Philips XL30, Amsterdam, Netherlands) and energy dispersive X-ray (EDX, integrated with SEM) to evaluate the microscopic features and chemical state as well as to analyze the progress of degradation after the experiments.

### 3.4. PDP Measurements

Mg specimens were fixed in a specimen holder at the bottom of a corrosion chamber adding 100 mL of MEM. The lower-side of the Mg specimens were mechanically connected to a working electrode, while its upper side was exposed to the simulated body fluid with a defined area of 1 cm^2^, as done in previous studies of our group [52]. Within the corrosion cell, a reference electrode (Ag/AgCl) and a counter electrode (graphite) were placed, providing an electrochemical three-electrode setup. The electrodes, forming a potentiostatic circuit, were placed into an incubator under physiological conditions (see above). Using a potentiostat (SP-150, Bio-Logic Science Instruments SAS, Seyssinet-Pariset, France), the open circuit potential, the electrochemical potential reached by a redox reaction in dynamic equilibrium was measured for 45 min before subjecting the specimens to potentiondynamic measurements at a range of −400 mV to +400 mV in reference to the OCP. The resulting Tafel plot was used to calculate the corrosion potential (V_corr_) and current (I_corr_) at the intersection of the tangent lines to the anodic and cathodic branches using the Bio-Logic EC-Lab V10.31 software. Degradation rates in µm/year were derived equally using the Stern and Geary equation as previously described [51].

### 3.5. Cytocompatibility and LIVE/DEAD Staining

For the cytocompatibility experiments, L-929-fibroblasts (ATCC, Wesel, Germany; obtained from the Department of Osteology and Biomechanics, University Medical Center Hamburg Eppendorf, Hamburg, Germany, 16 February 2013) were used. Cell culture was performed under physiological conditions, as described in 2.1. Cell passaging was accomplished at 80% confluence every 24 h according to EN ISO 109983-5/-12.

For direct testing, specimens and reference materials (negative control, positive control, glass control) were sterilized in isopropanol for 5 min and seeded with 2.4 × 10^5^ cells in 1.88 mL cell culture medium in 12 well plates. Cells were seeded directly onto the surface of the materials. Assays were carried out after 24 h incubation under cell culture conditions. XTT-, BrdU- and LDH assays were performed as previously described and according to the manufacturer’s instructions [50].

For live/Dead staining, 60 µL per ml medium propidium iodide (PI) stock solution (50 µg/mL in PBS) and 500 µL per mL medium fluorescein diacetate (FDA) working solution (20 µg/mL in PBS from 5 mg/mL FDA in acetone stock solution) was added to each well (12 well plate). After a brief incubation for 3 min at room temperature, specimens were rinsed in prewarmed PBS and were immediately examined with an upright fluorescence microscope (Nikon ECLIPSE Ti-S/L100, Nikon GmbH, Düsseldorf, Germany) equipped with a filter for parallel detection of red and green fluorescence. Images were taken at 4-fold, 10-fold, and 20-fold magnification.

### 3.6. Statistical Analysis

Data are expressed as means ± standard deviation. The Software GraphPad Prism (Version 7.05, San Diego, CA, USA) and two-way ANOVA were used to test for significant differences between the groups and time points. *p*-values of ≤ 0.05 (*) and ≤ 0.01 (**) were considered as statistically significant.

## 4. Discussion

The goal of this study was to design a dynamic testing setup for the in-vitro measurement of magnesium degradation by H_2-_evolution measurement and thus mimicking/simulating the in-vivo degradation behavior under physiological conditions. Further objectives were to demonstrate a proof-of-concept by subjecting unprotected as well as surface modified magnesium specimens to the experimental setup and measure the difference in degradation behavior over an exaggerated time period.

The fluid dynamic testing was established by considering physiological body temperature (37 °C), a corrosive medium similar to the human blood plasma (MEM + 10% FCS and Glutamine) and choosing a reasonable methodical approach to determine the degradation of the magnesium by measuring the evolving H_2_-volumes.

Studies have shown that the choice of a corrosive medium can have a major impact on the degradation rate [30,37,38,39,40,41,42,43,44,45]. For example, components, such as 4-2-hydroxyethyl-1-piperazineethanesulfonic acid (HEPES), vitamins, high concentrations of chloride and amino acids can act as corrosion accelerators, while the addition of bicarbonate (HCO_3_−) at concentrations of 27 mM/, H_2_PO_4_− and Albumin can slow down corrosions rates [43,53,54,55]. In our opinion, regardless of the composition used, the utilized medium should be as similar as possible to human blood plasma, prevent bacterial contamination, be affordable and easy to produce or source in order to ensure ease of use and comparability with other studies [50,56]. MEM, supplemented with 10% FCS, 10 U/mL penicillin/streptomycin and 4 mM *L*-glutamine, was met those requirements and was hence used in this study. The corrosion cells used for the testing within our study were installed in an incubator to guarantee physiological conditions at 37 °C, 6% CO_2_ and 100% humidity. Former studies investigated and implemented methods to measure magnesium degradation by using either static measurement methods, non-physiological measurement methods (PDP, SEM, pH) or environmental modalities offside from physiological conditions, e.g., corrosion mediums like saline solution [24,25,26,27,28,29,30,31,32,33,34,35,36,37,38,39,40,41,42,43,44,45]. Especially using static immersion testing to evaluate the degradation behavior can lead to falsified measurements when testing magnesium specimens. The rather high degradation rates of this material class usually lead to the accumulation of magnesium ions in the corrosion medium and to a severe shift in pH. Former studies facilitating static immersion testing have tried to compensate pH levels by either a recurring exchange of the corrosion medium and/or the use of pH buffers. However, most studies lack a frequent monitoring of the pH within the corrosion medium and corresponding results indicate the occurrence of pH shifts towards an alkaline milieu. While the human body is able to maintain the pH in the tissue surrounding an implant at least to a certain extent, this unphysiological conditioning during static testing not only shows to by unphysiological, but also do decrease the degradation rates of magnesium and thus distort the measurement in terms of in-vivo comparison. For this reason, we chose to facilitate dynamic single corrosion cell testing. Despite higher efforts to setup the experimental procedure, e.g., sterilization, flushing and installation of pumps, tubes and other components, we were thus able to keep the pH on an almost constant level within the corrosion cells. Due to the continuous exchange of the corrosion medium within the cells physiological conditions could be achieved and the physiological limits for pH levels were not exceeded during testing.

By mimicking the in vivo situation as closely as possible, it is important to correlate in vitro results with in vivo results. Therefore, we propose the use of previously described physiological modalities, a constant measurement of H_2_ gas evolutionrates as well as pH monitoring under dynamic flow conditions, which should further be complemented by other methods as, PDP, SEM or cytocompatibility assessmentsin order to allow for a comprehensive analysis and validation of the testing results.

Consequently, H_2_-derived degradation rates of not only unprotected Mg WE43 specimens, but also PEO treated samples (PEO-A and PEO-B) were determined and the results were correlated with other methods (cytocompatibility, SEM, pH measurement).

Our findings show, that these ceramized samples showed a significantly improved degradation behavior in terms of a reduced H_2_-release, when compared to the untreated test sample. This reduction in degradation rate could also be confirmed by pH measurements and SEM images of the according surface topographies, hence validating the experimental dynamic setup and providing a proof-of-concept. As measurements under static conditions showed to be invalid in previous studies, no comparison to static immersion testing was included in this study [57].

The examination time of 100 h showed to be limited by bacterial and fungal contamination. This limitation could be solved by increasing the concentration of antibacterial agents or adding antimycotic supplements to the corrosion medium. Furthermore, the installation not only of the bioreactor, but also the whole environment under sterile conditions, e.g., in a laminar flow bench, could potentially improve conditions and hence allow for further elongated testing periods. The relatively high standard deviations of the Mg WE43 H_2_-release in our study can mainly be explained by the corrosion mechanisms of untreated magnesium samples, where minor surface damages or impurities lead to increased pittings and cracks on the surface, and thus leading to an stochastical and exponential increase of the H_2_ release [11,17,18].

Our test bench simulates the degradation of magnesium materials under unloaded conditions. However, the introduction of loading as shown by Koo and Yang et al. would have a beneficial impact on the overall degradation rate, thereby representing a meaningful addition to the facilitated testing setup [38,39].

Furthermore, as shown in previous studies by our group, magnesium corrosion products can interfere with certain chemical agents of differents cell culture assays to determinine cytocompatibility [50,52]. Thus, it is mandatory to adjust the level of interference by incubation of the magnesium specimens without cells in order to determine the overall interference levels. In this context, the interference of the corrosion products of different magnesium alloys and their interference on cytocompatibility and cytotoxicity assays should be investigated [58]. Furthermore, more research should be done concerning the influence of pH changes and corrosion products of magnesium on the overall cell survival in cell culture testing [59].

Further studies regarding the development of degradation test benches to evaluate the degradation of magnesium alloys should, above all, precisely correlate the in vivo-degradation of magnesium alloys with the in vitro-test situation. Beside different load-bearing and not load-bearing implantation areas, the choice of an adequate animal model should focus on large animals and the proximity of the model to human conditions.

## 5. Conclusions

In order to establish a magnesium testing setup that efficiantly approximates in-vivo degradation of bioabsorbable magnesium, a fluid-dynamic bench setup with H_2_-detection under physiological conditions was established and validated by measuring the degradation behavior of untreated and PEO treated magnesium WE43. The facilitated setup showed to be a promising method to in vitro simulate in vivo conditions by maintaining the pH level in the corrosion cells within physiological boundaries. PEO treated specimens exhibited a significantly improved (lower) degradation rate compared to the untreated magnesium reference. Other methods as electrochemical measurements (PDP), monitoring of the pH levels and cytocompatibility cell culture testing were conducted complementary and confirmed these results. To mimic the in vivo situation more accurately, further investigations should consider the influence of mechanical load and the corrosion medium on the degradation behavior and aim to further increase the maximum duration of magnesium corrosion experiments by improving sterile conditions.

## Figures and Tables

**Table 1:** Calculated degradation of the Mg WE43 specimens with and without PEO coating in µm/year. The results show a manifold higher degradation rate for the untreated MgWe43 samples compared to the PEO modified samples. In concordance with the hydrogen gas production measured in the bioreactor system, PEO-B shows the lowest degradation rate.

Sample	Degradation Per Year/µm
Mg WE43	118
PEO-A	5
PEO-B	3

**Table 2:** Magnesium variants used in the experiments. PEO, plasma electrolytic oxidized.

Variant	Name	Brand Name	Composition
A	PEO-A	-	phosphate + potassium hydroxide
B	PEO-B	Kermasorb^®^	Phosphate + ammonium hydroxide

## References

[1] Chen Y., Xu Z., Smith C., Sankar J. (2014). Recent advances on the development of magnesium alloys for biodegradable implants. Acta Biomater..

[2] Staiger M.P., Pietak A.M., Huadmai J., Dias G. (2006). Magnesium and its alloys as orthopedic biomaterials: A review. Biomaterials.

[3] Lu H.H., Thomopoulos S. (2013). Functional attachment of soft tissues to bone: Development, healing, and tissue engineering. Annu. Rev. Biomed. Eng..

[4] Ge Z., Yang F., Goh J.C., Ramakrishna S., Lee E.H. (2006). Biomaterials and scaffolds for ligament tissue engineering. J. Biomed. Mater. Res. A.

[5] Castellani C., Lindtner R.A., Hausbrandt P., Tschegg E., Stanzl-Tschegg S.E., Zanoni G., Beck S., Weinberg A.M. (2011). Bone-implant interface strength and osseointegration: Biodegradable magnesium alloy versus standard titanium control. Acta Biomater..

[6] Chaya A., Yoshizawa S., Verdelis K., Myers N., Costello B.J., Chou D.T., Pal S., Maiti S., Kumta P.N., Sfeir C. (2015). In vivo study of magnesium plate and screw degradation and bone fracture healing. Acta Biomater..

[7] Glenske K., Donkiewicz P., Kowitsch A., Milosevic-Oljaca N., Rider P., Rofall S., Franke J., Jung O., Smeets R., Schnettler R. (2018). Applications of metals for bone regeneration. Int. J. Mol. Sci..

[8] Saleh M.M., Touny A.H., Al-Omair M.A. (2016). Biodegradable/biocompatible coated metal implants for orthopedic applications. Biomed. Mater. Eng..

[9] Farraro K.F., Kim K.E., Woo S.L., Flowers J.R., McCullough M.B. (2014). Revolutionizing orthopaedic biomaterials: The potential of biodegradable and bioresorbable magnesium-based materials for functional tissue engineering. J. Biomech..

[10] Zhao D., Witte F., Lu F., Wang J., Li J., Qin L. (2017). Current status on clinical applications of magnesium-based orthopaedic implants: A review from clinical translational perspective. Biomaterials.

[11] Wolf F.I., Cittadini A. (2003). Chemistry and biochemistry of magnesium. Mol. Aspects Med..

[12] Jahnen-Dechent W., Ketteler M. (2012). Magnesium basics. Clin. Kidney J..

[13] Saris N.E., Mervaala E., Karppanen H., Khawaja J.A., Lewenstam A. (2000). Magnesium. An update on physiological, clinical and analytical aspects. Clin. Chim. Acta.

[14] Noviana D., Paramitha D., Ulum M.F., Hermawan H. (2016). The effect of hydrogen gas evolution of magnesium implant on the postimplantation mortality of rats. J. Orthop. Translat..

[15] Schneider L.A., Korber A., Grabbe S., Dissemond J. (2007). Influence of pH on wound-healing: A new perspective for wound-therapy?. Arch. Dermatol. Res..

[16] Mueller W.D., Lucia Nascimento M., Lorenzo de Mele M.F. (2010). Critical discussion of the results from different corrosion studies of Mg and Mg alloys for biomaterial applications. Acta Biomater..

[17] Thomas S., Medhekar N.V., Frankel G.S., Birbilis N. (2015). Corrosion mechanism and hydrogen evolution on Mg. Curr. Opin. Solid State Mater. Sci..

[18] Song A., Atrens A. (2003). Understanding magnesium corrosion-A framework for improved alloy performance. Adv. Eng. Mater..

[19] Willbold E., Gu X., Albert D., Kalla K., Bobe K., Brauneis M., Janning C., Nellesen J., Czayka W., Tillmann W. (2015). Effect of the addition of low rare earth elements (lanthanum, neodymium, cerium) on the biodegradation and biocompatibility of magnesium. Acta Biomater..

[20] Yang J., Cui F., Lee I.S. (2011). Surface modifications of magnesium alloys for biomedical applications. Ann. Biomed. Eng..

[21] Wang J., Tang J., Zhang P., Li Y., Lai Y., Qin L. (2012). Surface modification of magnesium alloys developed for bioabsorbable orthopedic implants: A general review. J. Biomed. Mater. Res. B: Appl. Biomater..

[22] Tian P., Liu X. (2015). Surface modification of biodegradable magnesium and its alloys for biomedical applications. Regen. Biomater..

[23] Hornberger H., Virtanen S., Boccaccini A.R. (2012). Biomedical coatings on magnesium alloys a review. Acta Biomater..

[24] Persaud-Sharma D., Budiansky N. (2013). In vitro degradation behavior of ternary Mg-Zn-Se and Mg-Zn-Cu alloys as biomaterials. J. Biomim. Biomater. Tissue Eng..

[25] Pardo A., Celiu Jr. S., Mercino R., Arrabal R., Matykina E. (2010). Electrochemical estimation of the corrosion rate of Magnesium/Aluminium alloys. Int. J. Corros..

[26] Kannan M.B., Raman R.K. (2008). In vitro degradation and mechanical integrity of calcium-containing magnesium alloys in modified-simulated body fluid. Biomaterials.

[27] Gu X.N., Zheng Y.F., Chen L.J. (2009). Influence of artificial biological fluid composition on the biocorrosion of potential orthopedic Mg-Ca, AZ31, AZ91 alloys. Biomed. Mater..

[28] Wolters L., Besdo S., Angrisani N., Wriggers P., Hering B., Seitz J.M., Reifenrath J. (2015). Degradation behaviour of LAE442-based plate-screw-systems in an in vitro bone model. Mater. Sci. Eng. C.

[29] Klein M., Walther F. (2016). Electrochemical-controlled characterization of the corrosion fatigue behavior of creep-resistant Magnesium alloys DieMag422 and AE42-ScienceDirect. Procedia Eng..

[30] Kirkland N.T., Birbilis N., Staiger M.P. (2012). Assessing the corrosion of biodegradable magnesium implants: A critical review of current methodologies and their limitations. Acta Biomater..

[31] Fajardo S., Frankel G.S. (2015). Gravimetric method for hydrogen evolution measurements on dissolving Magnesium. J. Electrochem. Soc..

[32] Curioni M. (2014). The behaviour of magnesium during free corrosion and potentiodynamic polarization investigated by real-time hydrogen measurement and optical imaging. Electrochim. Acta.

[33] Birbilis N., King A.D., Thomas S., Frankel G.S., Scully J.R. (2014). Evidence for enhanced catalytic activity of magnesium arising from anodic dissolution. Electrochem. Acta.

[34] Ascencio M., Pekguleryuz M., Omanovic S. (2015). Correlation between electrochemical impedance measurements and corrosion rate of magnesium investigated by real-time hydrogen measurement and optical imaging. Electrochim. Acta.

[35] Witte F., Fischer J., Nellesen J., Crostack H.A., Kaese V., Pisch A., Beckmann F., Windhagen H. (2006). In vitro and in vivo corrosion measurements of magnesium alloys. Biomaterials.

[36] Liu C.L., Wang Y.J., Zeng R.C., Zhang X.M., Huang W.J., Chu P.K. (2010). In vitro corrosion degradation behaviour of Mg-Ca alloy in the presence of albumin. Corros. Sci..

[37] Shadanbaz S., Walker J., Staiger M.P., Dias G.J., Pietak A. (2013). Growth of calcium phosphates on magnesium substrates for corrosion control in biomedical applications via immersion techniques. J. Biomed. Mater. Res. B: Appl. Biomater..

[38] Koo Y., Lee H.B., Dong Z., Kotoka R., Sankar J., Huang N., Yun Y. (2017). The effects of static and dynamic loading on biodegradable magnesium pins in vitro and in vivo. Sci. Rep..

[39] Yang G.F., Kim Y.C., Han H.S., Lee G.C., Seok H.K., Lee J.C. (2015). In vitro dynamic degradation behavior of new magnesium alloy for orthopedic applications. J. Biomed. Mater. Res. B: Appl. Biomater..

[40] Zheng F., Rassat S.D., Helderandt D.J., Caldwell D.D., Aardahl C.L., Autrey T., Linehan J.C., Rappe K.G. (2008). Automated gas burette system for evolved hydrogen measurements. Rev. Sci. Instrum..

[41] Song G., Atrens A., StJohn D. (2011). An hydrogen evolution method for the estimation of the corrosion rate of magnesium alloys. Magnes. Technol..

[42] Nidadavolu E.P.S., Feyerabend F., Ebel T., Willumeit-Romer R., Dahms M. (2016). On the determination of magnesium degradation rates under physiological conditions. Mater. (Basel.).

[43] Walker J., Shadanbaz S., Kirkland N.T., Stace E., Woodfield T., Staiger M.P., Dias G.J. (2012). Magnesium alloys: Predicting in vivo corrosion with in vitro immersion testing. J. Biomed. Mater. Res. B: Appl. Biomater..

[44] Frankel G.S., Samaniego A., Birbilis N. (2013). Evolution of hydrogen at dissolving magnesium surfaces. Corros. Sci..

[45] Frankel G.S., Fajardo S., Lynch B.M. (2015). Introductory lecture on corrosion chemistry: A focus on anodic hydrogen evolution on Al and Mg. Faraday Discuss..

[46] Gonzalez J., Hou R.Q., Nidadavolu E.P.S., Willumeit-Romer R., Feyerabend F. (2018). Magnesium degradation under physiological conditions-Best practice. Bioact. Mater..

[47] Reifenrath J., Marten A.K., Angrisani N., Eifler R., Weizbauer A. (2015). In vitro and in vivo corrosion of the novel magnesium alloy Mg-La-Nd-Zr: Influence of the measurement technique and in vivo implant location. Biomed. Mater..

[48] Agha N.A., Feyerabend F., Mihailova B., Heidrich S., Bismayer U., Willumeit-Romer R. (2016). Magnesium degradation influenced by buffering salts in concentrations typical of in vitro and in vivo models. Mater. Sci. Eng. C.

[49] Witecka A., Bogucka A., Yamamoto A., Mathis K., Krajnak T., Jaroszewicz J., Swieszkowski W. (2016). In vitro degradation of ZM21 magnesium alloy in simulated body fluids. Mater. Sci. Eng. C.

[50] Jung O., Smeets R., Porchetta D., Kopp A., Ptock C., Muller U., Heiland M., Schwade M., Behr B., Kroger N. (2015). Optimized in vitro procedure for assessing the cytocompatibility of magnesium-based biomaterials. Acta Biomater..

[51] Ascencio M., Pekguleryuz M., Omanovic S. (2014). An investigation of the corrosion mechanisms of WE43 Mg alloy in a modified simulated body fluid solution: The influence of immersion time. Corros. Sci..

[52] Jung O., Smeets R., Hartjen P., Schnettler R., Feyerabend F., Klein M., Wegner N., Walther F., Stangier D., Henningsen A. (2019). Improved in vitro test procedure for full assessment of the cytocompatibility of degradable magnesium based on ISO 10993-5/-12. Int. J. Mol. Sci..

[53] Mueller W.D., de Mele M.F., Nascimento M.L., Zeddies M. (2009). Degradation of magnesium and its alloys: Dependence on the composition of the synthetic biological media. J. Biomed. Mater. Res. A.

[54] Xin Y., Hu T., Chu P.K. (2011). In vitro studies of biomedical magnesium alloys in a simulated physiological environment: A review. Acta Biomater..

[55] Ng W.F., Chiu K.Y., Cheng F.T. (2010). Effect of pH on the in vitro corrosion rate of magnesium degradable implant material. Mater. Sci. Eng. C.

[56] Jung O., Smeets R., Kopp A., Porchetta D., Hiester P., Heiland M., Friedrich R.E., Precht C., Hanken H., Grobe A. (2016). PEO-generated surfaces support attachment and growth of cells in vitro with no additional benefit for micro-roughness in Sa (0.2-4 mum). in vivo.

[57] Wang H., Shi Z. (2011). In vitro biodegradation behavior of magnesium and magnesium alloy. J. Biomed. Mater. Res. B: Appl. Biomater..

[58] Jang Y., Collins B., Sankar J., Yun Y. (2013). Effect of biologically relevant ions on the corrosion products formed on alloy AZ31B: An improved understanding of magnesium corrosion. Acta Biomater..

[59] Keim S., Brunner J.G., Fabry B., Virtanen S. (2011). Control of magnesium corrosion and biocompatibility with biomimetic coatings. J. Biomed. Mater. Res. B: Appl. Biomater..

